# Effect of phosphorus supplementation on weight gain and waist circumference of overweight/obese adults: a randomized clinical trial

**DOI:** 10.1038/nutd.2015.38

**Published:** 2015-12-21

**Authors:** J J Ayoub, M J A Samra, S A Hlais, M S Bassil, O A Obeid

**Affiliations:** 1 Department of Nutrition and Food Science, Faculty of Agricultural and Food Sciences, American University of Beirut, Beirut, Lebanon; 2 Department of Family Medicine, Faculty of Medicine, American University of Beirut Medical Center, Beirut, Lebanon; 3 Department of Natural Sciences, Faculty of Arts and Sciences, Lebanese American University, Beirut, Lebanon

## Abstract

**Background:**

Phosphorus status is inversely correlated with body weight; however, the effect of phosphorus supplementation on body weight in a controlled design has not been studied.

**Methods:**

This is a double-blind, randomized, placebo-controlled trial of 63 adults aged 18–45 years with a body mass index (BMI) of ⩾25 kg m^−2^ and normal kidney function at the American University of Beirut. Participants were randomly assigned to the placebo or phosphorus group where daily placebo or phosphorus supplements were ingested with three main meals (breakfast, lunch and dinner) for a period of 12 weeks. Primary outcomes were changes in anthropometric measures, blood metabolites (including lipid profile, glucose and insulin) and subjective appetite scores. The trial is registered with Clinical Trial.gov, NCT02329990.

**Results:**

Body weight was significantly lower in the phosphorus group when compared with the placebo group (−0.65 kg (95% confidence interval (CI) −1.69 to 0.40) vs 1.13 kg (95% CI 0.19 to 2.06), *P*=0.01). Similarly, BMI and waist circumference were significantly lower in the phosphorus group when compared with the placebo group (−0.24 kg m^−2^ (95% CI −0.59 to 0.12) vs 0.42 kg m^−2^ (95% CI 0.05 to 0.78), *P*=0.01; −3.62 cm (95% CI−4.90 to −2.33) vs 0.38 cm ( 95% CI−0.44 to 1.20), *P*<0.001; respectively). Several parameters of subjective appetite scores were decreased in the phosphorus-supplemented group.

**Conclusions:**

Phosphorus supplementation for 12 weeks significantly decreases body weight, BMI, waist circumference and subjective appetite scores. These findings support a promising role of the mineral phosphorus in the prevention and management of obesity, especially abdominal adiposity. The exact mechanisms of action and longer-term effects still need to be elucidated.

## Introduction

Obesity is increasing at alarming rates in many high-, medium- and low-income countries.^1^ This is contributing to the development of many metabolic diseases, including diabetes and cardiovascular disease.^2^

Modernization, including food industrialization and globalization of food markets, has been correlated with the increased consumption of products containing negligible amounts of phosphorus such as refined cereals (whereby refinement reduces phosphorus content by ∼70%), oils, sugars and sweeteners that are currently contributing to >50% of the food supply (kcal per capita per day) in most countries.^3^ This has caused a decrease in daily phosphorus ingestion to ∼1–1.5 g day^−1^,^4, 5^ as compared with our ancestors' estimated intake of 2.5 g day^−1^ (based on primarily raw, unprocessed foods with a 2500 kcal day^−1^ diet and ∼1 mg phosphorus per kcal).^3^

Low phosphorus status has been positively associated with increased body weight.^3, 6, 7^ This may be attributed to the impact of hepatic adenosine triphosphate (ATP), which depends on adequate dietary supply of phosphorus, on suppressing food intake.^3, 8, 9^ This mechanism is supported by an inverse relation between body weight and hepatic ATP status.^10, 11, 12^ In line with that, we have previously found that phosphorus addition to carbohydrate preloads significantly reduces *ad libitum* energy intake at subsequent meal.^13^

Given the increased prevalence of obesity among individuals consuming abundant quantities of food containing low levels of phosphorus, it is reasonable to postulate that decreased phosphorus intake may be involved in the development of obesity and its metabolic abnormalities. Hence, we conducted a randomized, placebo-controlled trial to examine the effects of 12-week phosphorus supplementation on body weight, body mass index (BMI), waist circumference and subjective appetite scores in overweight and obese adults.

## Materials and methods

### Participants

**Figure 1:**
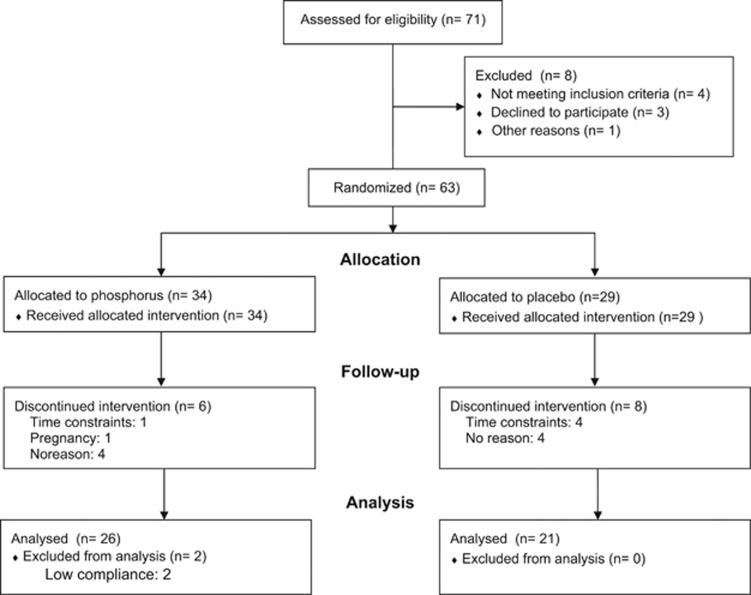
Study flow diagram.

After approval of the study by the institutional review board at the American University of Beirut (Beirut, Lebanon), 63 adults aged 18 to 45 years with a BMI ⩾25 kg m^−2^, who provided signed informed consent, were recruited from the general public using poster advertisements or direct approach. Details about recruitment, randomization and follow-up are presented in Figure 1. Exclusion criteria included glomerular filtration rate <60 ml min^−1^ per 1.73 m^2^, presence of any significant medical disease, pregnancy or lactation, regular administration of drugs that affect body weight and weight change of ⩾3% within 3 months before the study. The study was conducted between June 2013 and September 2014 at the American University of Beirut. We computed that the enrollment of 40 subjects (20 per group) would detect a 10% change in weight of the placebo group, assuming the latter having a mean weight of 90 kg and s.d. of 10 kg, with 80% power and an α of 5%. The trial is registered with Clinical Trial.gov, NCT02329990.

### Randomization and masking

This double-blind, randomized, controlled study allocated subjects into placebo group (*n*=21) or phosphorus group (*n*=26). Participants were requested to take three tablets containing either 375 mg phosphorus or a placebo (Nutricap Labs, Farmingdale, NY, USA) with each main meal (breakfast, lunch and dinner) for 12 weeks. They were asked to maintain regular dietary and physical activity habits during the entire study course and avoid alcohol consumption and any strenuous exercise 24 h before their visits (at baseline, 6 weeks and 12 weeks). Assignment to intervention or control group was made by having the principle investigator (corresponding author) ask the eligible subjects to blindly draw an envelope from a large box of 100 opaque, sealed envelopes (50 for each group), each containing a 2-cm by 2-cm paper with a written code designating intervention or control. There were no detectable differences in size or weight between intervention and control envelopes. In addition, both researchers and participants were blinded for the type of supplements that were similar in size, shape, color and odor.

### Procedures

Subjects were asked to attend the research unit at baseline and after 6 and 12 weeks of participation. At baseline, anthropometric measurements and blood samples were collected and a subjective appetite questionnaire based on Wilson *et al.*^14^ was completed. Participants were given a 6-week supply of the allocated supplement and were asked to attend the research unit at the end of this period. At 6 weeks, remaining tablets were collected and counted in order to assess adherence to the allocated intervention. Participants were then given a supply of the same type of supplementation for the consequent 6 weeks and were asked to complete the subjective appetite questionnaire. At 12 weeks, data were collected similar to the baseline visit, and remaining tablets were counted to assess compliance. Individuals who consumed >70% of the allocated tablets were excluded.

Body weight and height (without shoes) were measured to the nearest 0.1 kg and 0.1 cm, using a calibrated Seca balance (Hamburg, Germany) and a portable stadiometer, respectively. Blood was withdrawn after overnight fast and samples were centrifuged for 15 min at 3500 r.p.m. at 3 °C for serum and plasma separation. Sample aliquots were stored at −80 °C until analysis. Serum phosphorus, creatinine, C-reactive protein, total cholesterol, high-density lipoprotein cholesterol, triglyceride and glucose levels were measured using the Vitros 350 analyzer (Ortho Clinical Diagnostics, Johnson and Johnson, Buckinghamshire, UK). The Friedwald formula^15^ was used to calculate low-density lipoprotein cholesterol levels. Fasting insulin concentration was measured using the ELISA kit (Diametra Millipore, Billerica, MA, USA). HOMA-IR (homeostasis model assessment of insulin resistance) was calculated as described by Matthews *et al.*^16^ Glomerular filtration rate was calculated using CKD-EPI (Chronic Kidney Disease Epidemiology Collaboration) estimated glomerular filtration rate.^17^

### Statistical analysis

Pairwise changes from baseline to 12-week follow-up anthropometric and biochemical variables were tested using paired *t-*tests, and intergroup assessment was performed using two-sample *t-*test. Repeated measures analysis of variance test was applied to analyze intragroup variation of appetite scores at different periods of time (baseline, 6 weeks and 12 weeks). Statistical analyses were conducted using SPSS 22 (Chicago, IL, USA).

### Role of the funding source

The study was funded by the National Council for Scientific Research, Lebanon. The funding source had no role in the study design or conduct; data collection, analysis, interpretation or reporting of the data; preparation, review or approval of the manuscript; or decision to submit the manuscript for publication.

## Results

### Subject characteristics

Baseline characteristics are shown in Table 1, and they were similar between groups. In all, 47 participants (placebo group *n*=21; phosphorus group *n*=26) completed the intervention, and all subjects had normal glomerular filtration rate (>60 ml min^−1^ per 1.73 m^2^) with a mean of 114.14 (10.19) ml min^−1^ per 1.73 m^2^ and 112.24 (13.46) ml min^−1^ per 1.73 m^2^ for the placebo and phosphorus groups, respectively. The mean age was 36.67 (9.76) years in the placebo group and 34.04 (11.24) years in the phosphorus group. No side effects were reported by participants over the experimental period.

### Anthropometric assessments

Changes in anthropometric and biochemical characteristics from baseline to 12 weeks are shown in Table 2. Body weight of the placebo group increased significantly from baseline by 1.13 kg (95% confidence interval (CI) 0.19 to 2.06), whereas that of the phosphorus group decreased by 0.65 kg (95% CI −1.69 to 0.40). These variations resulted in a significant difference (*P*=0.01) in the changes in body weight between the two groups. Consequently, the changes in BMI of the placebo group (0.42 kg m^−2^, 95% CI 0.05 to 0.78) was significantly different (*P*=0.01) than that of the phosphorus group (−0.24 kg m^−2^, 95% CI −0.59 to 0.12). Simultaneously, waist circumference of the phosphorus group was significantly reduced by 3.62 cm (95% CI −4.90 to −2.33), and this reduction was significantly different (*P*<0.001) from the small increase of 0.38 cm (95% CI −0.44 to 1.20) in the waist circumference of the placebo group.

### Biochemical assessments

Placebo or phosphorus treatment for 12 weeks did not affect serum levels of phosphorus, total cholesterol, low-density lipoprotein cholesterol, high-density lipoprotein cholesterol, triglyceride, glucose and C-reactive protein. Serum levels of insulin and HOMA-IR were similar between the two treatments at baseline and at 12 weeks, although a mild but significant difference was detected in their changes. This mild change is not believed to be of clinical significance (Table 2).

### Subjective appetite scores

Baseline subjective appetite scores were similar between groups. The changes in several parameters of subjective appetite scores were found to decrease as the experiment progressed including that of appetite, quantity of food to reach fullness, hunger and number of snacks. However, changes in appetite, quantity of food to reach fullness, taste of food and number of snacks were significantly reduced in the phosphorus group as compared with the placebo as shown in Table 3.

## Discussion

Several dietary patterns and interventions were reported to improve body weight.^18, 19, 20, 21, 22^ High protein diets were constantly found to induce weight loss, probably because of their capacity to decrease energy intake and increase energy expenditure.^18, 23, 24^ Consumption of dairy products was also shown to be inversely related to body weight, whereby its increased intake among overweight individuals was reported to lower body weight,^19, 25^ irrespective of its calcium content.^26^ Moreover, the intake of whole grains was shown to be negatively associated with the risk of different components of the metabolic syndrome, including body weight;^20, 21^ however, the mechanism of such effect remains uncertain.^27^ This raises the questions on the role of macronutrients in weight reduction, especially as these dietary patterns or interventions have varied macronutrient profiles.^22^ The common feature between these diets seems to be their phosphorus content, as proteins, dairy products and whole grains are rich sources of phosphorus.^28^ This was the rationale for our proposed hypothesis on the involvement of low phosphorus status in the development of obesity and metabolic syndrome.^3, 29^

Our study found that the ingestion of 375 mg phosphorus with each main meal, over a period of 12 weeks, was able to prevent weight gain and to reduce waist circumference among overweight and obese adults. However, minimal alterations were observed in the measured biochemical parameters (lipid profile, glucose and so on) that may be attributed to the modest baseline abnormalities in these parameters, short experimental duration and/or to the modest anthropometric changes. The absence of change in fasting plasma phosphorus further confirms that it is not a good marker of phosphorus intake.^30^

The anthropometric changes in the phosphorus group are in line with other studies in which phosphorus status was reported to be inversely related to body weight^3, 6, 7, 29^ and waist circumference.^3, 31, 32^ The mechanism(s) by which phosphorus affected body weight may have been related to its involvement in food intake control and/or energy metabolism.^3^ Phosphorus availability is known to stimulate ATP production, in particular hepatic ATP,^8, 33^ that is believed to transmit afferent neural signals to the central nervous system resulting in a decrease in food intake^8^ through the stimulation of satiation. Such effect was believed to be behind the impact of phosphorus addition to different carbohydrate preloads on the suppression of *ad libitum* energy intake at subsequent meal.^13^ In agreement, as reported in the subjective appetite questionnaires, satiation indicated by the quantity of food to reach fullness was reduced in the phosphorus group; however, the number of main meals, which is an indicator of satiety, was not reduced. Sustenance of hepatic ATP production over the postprandial and postabsorptive periods may have contributed to the observed reduction in appetite and number of snacks and these may have been translated by subjects into taste changes. Conversely, the similarity in the scores of hunger (that is, physiological controlled by depletion of energy stores) and the number of main meals between the phosphorus and placebo groups may be explained by a limited availability of hepatic ATP substrates beyond postprandial and postabsorptive periods, and thus an inability to impact the initiation of the next main meal. In brief, the impact of phosphorus supplement on energy intake seems to be related to its capacity to reduce the size of main meals (low appetite and high fullness) as well as intake between meals (number of snacks).

Furthermore, the favorable differences in body weight and waist circumference in the phosphorus group may have been partially related to an effect of phosphorus on energy metabolism. The addition of phosphorus to orange juice was reported to increase postprandial thermogenesis among obese but not lean subjects.^34, 35^ In addition, phosphorus supplementation in a weight reducing program was found to increase resting metabolic rate of obese subjects.^36, 37^ The pronounced reduction in waist circumference in the face of the modest reduction in body weight may have been attributed to changes in body composition. Weight gain under phosphorus-deficient diet was reported to be largely attributed to an increase in adipose tissue, whereas nitrogen retention was impaired,^38^ and this seems to mimic that of low-protein (low-phosphorus) diet.^23^ Changes in body fat were reported to be related to energy intake, whereas changes in lean body mass were related to the intake of protein,^23^ known to be high in phosphorus and this raises a question of whether the effect of protein on weight gain is linked to its content of phosphorus. It is not clear whether phosphorus supplementation favored lean body mass retention that ultimately masked the effect on changes in body weight because of its capacity to retain water. In any case, the observed reduction in waist circumference was similar to that reported in subjects under low-fat diets,^22^ and is believed to be of clinical significance as it is an indicator of abdominal obesity (visceral fat) that is known to be a risk factor of type 2 diabetes and cardiovascular disease.^39, 40^

Many concerns were raised on the relation between phosphorus status and cardiovascular disease and mortality,^41, 42^ although the nature of the relation with phosphorus intake is far from clear and requires further scrutiny,^43^ especially as fasting serum phosphorus does not reflect intake^30, 44^ as confirmed by our results. The fact that fasting but not nonfasting (that reflects intake rather than clearance) serum phosphorus levels were associated with increased mortality^45^ and fasting serum phosphorus level but not dietary intake were associated with coronary artery calcification^46^ may imply that factors behind or associated with elevated fasting serum phosphorus rather than phosphorus intake may have attributed to these detrimental effects. The recent reported weak association between dietary phosphorus intake and all-cause mortality^44^ was questioned as varied dietary habits or profiles were seen among the different dietary phosphorus intake quartiles. Moreover, such association may have been cofounded by the source of phosphorus in the diet, especially as dietary heme iron intake (derived from animal source that is also high in phosphorus) was shown to increase the risk of cardiovascular disease.^47^ It is believed that the need of phosphorus especially for carbohydrate metabolism may have been compromised by modernization (refinement and so on), particularly in staple carbohydrate-rich foods (rice, wheat and so on). The impact of such a compromise is expected to depend on the contribution of staple food to total energy intake and may partially be behind the drastic increase in obesity in developing countries, in particular as carbohydrate contribution to total energy intake is inversely related to income.^48^

The weaknesses of the study include: the lack of biomarker for phosphorus intake, the use of the subjective self-reported visual analog scale (VAS) for the determination of appetite scores and the lack of analyses for body composition and energy expenditure.

The strength of our study was that a rigorous system of training and certification of study personnel was developed and implemented for collecting all data. In addition, our study is pragmatic, randomized, double blinded and placebo controlled that required the use of tablets without requesting behavioral or dietary changes to avoid the problem of adherence.

## Conclusion

In summary, this 12-week randomized, placebo-controlled trial showed that phosphorus supplementation (375 mg per main meal) halted weight gain and BMI while significantly decreasing waist circumference. This may have been related to favorable changes in body composition. At the same time, these changes were associated with early satiation, whereas satiety (number of main meals) and hunger were not altered. Our findings support a promising role of the mineral phosphorus in preventing obesity, especially abdominal adiposity. Therefore, after extensive investigations, phosphorus utilization could be considered for the future development of weight maintenance or reduction supplements or implementation of flour fortification. Additional research is warranted to examine the exact mechanisms of actions and longer-term effects of phosphorus.

## Figures and Tables

**Table 1:** Baseline characteristics of the study participants

*Baseline characteristics*	*Placebo group (*n*=21)*	*Phosphorus group (*n*=26)*	*Unpaired* t*-test,* P-*value*
Age, mean (s.d.), years	36.67 (9.76)	34.04 (11.24)	0.396
			
*Sex, no. (%)*			
Male	6 (28.57)	10 (38.46)	
Female	15 (71.43)	16 (61.54)	
			
*Anthropometric measurements, mean (s.d.)*			
Weight, kg	92.33 (14.99)	88.33 (19.14)	0.426
Height, m	1.65 (0.08)	1.66 (0.11)	0.646
BMI^a^	33.73 (3.84)	31.64 (4.64)	0.099
Waist circumference, cm^b^	109.43 (9.90)	106.00 (12.74)	0.305
			
*Biochemical characteristics, mean (s.d.)*			
Serum phosphorus, mg dl^−1^ c	79.99 (41.54)	91.50 (53.20)	0.540
Total cholesterol, mg dl^−1^ d	221.85 (40.66)	216.08 (42.32)	0.645
LDL-C, mg dl^−1^ d	143.29 (30.47)	145.77 (33.50)	0.797
HDL-C, mg dl^−1^ d	46.30 (10.98)	43.12 (11.86)	0.357
Triglycerides, mg dl^−1^ d	161.50 (67.60)	137.40 (81.20)	0.284
Glucose, mg dl^−1^ d	95.25 (10.56)	96.28 (11.37)	0.755
Insulin, μIU ml^−1^ d	11.41 (7.77)	7.71 (7.89)	0.123
HOMA-IR^d^	2.81 (2.30)	1.93 (2.27)	0.210
CRP, mg l^−1^ d	9.82 (5.51)	9.40 (3.93)	0.773
GFR (ml min^−1^ per 1.73 m^2^)^d^	114.14 (10.19)	112.24 (13.46)	0.592

Abbreviations: BMI, body mass index; CRP, C-reactive protein; GFR, glomerular filtration rate; HDL-C, high-density lipoprotein cholesterol; HOMA-IR, homeostatic model assessment of insulin resistance; LDL-C, low-density lipoprotein cholesterol.

SI conversion factor: to convert serum phosphorus to mmol l^−1^, multiply by 0.323; cholesterol, LDL-C and HDL-C to mmol l^−1^, multiply by 0.0259; triglycerides to mmol l^−1^, multiply by 0.0113; glucose to mmol l^−1^, multiply by 0.0555.

a Calculated as weight in kg divided by height in m squared.

b Measured at the midpoint between the lower rib and iliac crest.

c Because of missing data, based on sample size of 20 and 26 for placebo and phosphorus groups, respectively.

d Because of missing data, based on sample size of 20 and 25 for placebo and phosphorus groups, respectively.

**Table 2:** Changes in anthropometric and biochemical characteristics from baseline to 12 weeks

*Indicator*	*Placebo group*			*Phosphorus group*			P-*value*^a^*, placebo vs phosphorus*
	*Sample size*^b^	*Mean difference (95% CI)*	P-*value*^c^	*Sample size*^b^	*Mean difference (95% CI)*	P-*value*^c^	
*Anthropometric measurements*							
Weight, kg	21	1.13 (0.19 to 2.06)	0.02	26	−0.65 (−1.69 to 0.40)	0.22	0.01
BMI^d^	21	0.42 (0.05 to 0.78)	0.03	26	−0.24 (−0.59 to 0.12)	0.19	0.01
Waist circumference, cm^e^	21	0.38 (−0.44 to 1.20)	0.35	26	−3.62 (−4.90 to −2.33)	<0.001	<0.001
							
*Biochemical characteristics*							
Serum phosphorus, mg dl^−1^	20	0.163 (0.034 to 0.292)	0.82	26	−0.111 (−0.299 to 0.077)	0.78	0.017
Total cholesterol, mg dl^−1^	20	−1.00 (−12.83 to 10.83)	0.86	25	0.92 (−11.45 to 13.29)	0.88	0.82
LDL-C, mg dl^−1^	20	2.37 (−10.10 to 14.84)	0.70	25	1.43 (−8.71 to 11.58)	0.77	0.90
HDL-C, mg dl^−1^	20	0.95 (−1.57 to 3.47)	0.44	25	−0.04 (−3.59 to 3.51)	0.98	0.64
Triglycerides, mg dl^−1^	20	−20.30 (−41.70 to 1.20)	0.06	25	−2.92 (−21.66 to 15.82)	0.75	0.21
Glucose, mg dl^−1^	20	−1.35 (−5.97 to 3.27)	0.55	25	0.64 (−6.98 to 8.26)	0.86	0.65
Insulin, mg dl^−1^	20	−2.61 (−6.52 to 1.29)	0.18	25	2.01 (−0.56 to 4.58)	0.12	0.05
HOMA-IR	20	−0.77 (−1.89 to 0.35)	0.17	25	0.79 (−0.26 to 1.83)	0.13	0.04
CRP, mg dl^−1^	20	1.25 (−0.65 to 3.15)	0.19	25	0.18 (−1.68 to 2.04)	0.84	0.41

Abbreviations: BMI, body mass index; CI, confidence interval; CRP, C-reactive protein; HDL-C, high-density lipoprotein cholesterol; HOMA-IR, homeostatic model assessment of insulin resistance; LDL-C, low-density lipoprotein cholesterol.

SI conversion factor: to convert serum phosphorus to mmol l^−1^, multiply by 0.323; cholesterol, LDL-C and HDL-C to mmol l^−1^, multiply by 0.0259; triglycerides to mmol l^−1^, multiply by 0.0113; glucose to mmol l^−1^, multiply by 0.0555.

a *P-*value for intergroup comparisons using two-sample *t-*test.

b Based on analysis of patients for whom data were available.

c *P-*values for pairwise intragroup comparisons obtained using paired *t-*test.

d Calculated as weight in kg divided by height in m squared.

e Measured at the midpoint between the lower rib and iliac crest.

**Table 3:** Changes in subjective appetite scores from baseline to 12 weeks

*Variable*	*Group*	*Sample size (*n)	*Mean difference (95% CI) 6 weeks*	*Mean difference (95% CI) 12 weeks*	P-*value*^a^		
					*Time*	*Treatment*	*Time × treatment*
Appetite	Placebo	21	−0.33 (−0.77 to 0.11)	−0.24 (−0.67 to 0.19)	0.002	0.01	0.18
	Phosphorus	26	−0.92 (−1.38 to −0.47)	−0.73 (−1.24 to −0.23)			
Quantity of food to reach fullness	Placebo	21	−0.57 (−0.94 to −0.20)	−0.33 (−0.77 to 0.11)	<0.001	0.04	0.30
	Phosphorus	26	−0.85 (−1.24 to −0.46)	−0.81 (−1.25 to −0.37)			
Hunger	Placebo	21	−0.62 (−1.09 to −0.15)	−0.33 (−0.75 to 0.08)	<0.001	0.31	0.53
	Phosphorus	26	−0.73 (−1.15 to −0.31)	−0.73 (−1.12 to −0.34)			
Taste of food	Placebo	21	0.00 (−0.20 to 0.20)	−0.05 (−0.32 to 0.22)	0.09	0.007	0.16
	Phosphorus	26	−0.31 (−0.56 to −0.06)	−0.35 (−0.60 to −0.09)			
Number of main meals	Placebo	21	0.14 (−0.08 to 0.36)	0.19 (0.01 to 0.37)	0.76	0.59	0.90
	Phosphorus	26	0.08 (−0.47 to 0.62)	0.04 (−0.50 to 0.58)			
Number of snacks	Placebo	21	−1.90 (−0.61 to 0.23)	−0.10 (−0.55 to 0.36)	0.04	0.01	0.21
	Phosphorus	26	−0.81 (−1.33 to −0.28)	−0.81 (−1.39 to −0.23)			

Abbreviation: CI, confidence interval.

a *P-*values for repeated measures analysis of variance (ANOVA).
